# The effect of body mass index on thoracic paravertebral block analgesia after video-assisted thoracoscopic surgery; a prospective interventional study

**DOI:** 10.1186/s12871-023-02264-0

**Published:** 2023-09-04

**Authors:** Emine Nilgün Zengin, Ali Alagöz, Hülya Yiğit, Hilal Sazak, Sumru Şekerci, Musa Zengin

**Affiliations:** 1 Ministry of Health Ankara Bilkent City Hospital, Anesthesiology and Reanimation Clinic, Ankara, Turkey; 2 University of Health Sciences, Ankara Atatürk Sanatorium Training and Research Hospital, Ankara, Turkey; 3 Ministry of Health Ankara Etlik City Hospital, Anesthesiology and Reanimation Clinic, Ankara, Turkey

**Keywords:** Acute pain, Body mass index, Paravertebral block, Patient-controlled analgesia, Postoperative analgesia, Video-assissted thoracoscopic surgery

## Abstract

**Background:**

To investigate the effects of body mass index (BMI) on intensity postoperative pain in patients who underwent thoracic paravertebral block (TPVB) for postoperative analgesia after video-assissted thoracoscopic surgery (VATS).

**Methods:**

Patients aged 18–80 years, ASA I-III, and BMI 18–40 kg/m^2^ who underwent elective VATS were included in the study. The patients were divided into 3 groups according to their BMI levels. TPVB was performed under ultrasound-guidance at the fifth thoracic vertebrae, and 30 ml of 0.25% bupivacaine was injected. The patient-controlled analgesia (PCA) was performed by using morphine and multimodal analgesia was performed. As a rescue analgesic agent, 0.5 mg/kg tramadol was given to patients intravenously when a score of visual analog scale (VAS) at rest was ≥ 4. The primary outcome was determined as VAS scores at rest and cough. Secondary outcomes were determined as postoperative morphine consumption, additional analgesic requirement, and side effects.

**Results:**

The post-hoc test revealed that the VAS resting scores at the 4^th^ hour (p: 0.007), 12^th^ hour (p: 0.014), and 48^th^ hour (p: 0.002) were statistically significantly lower in Group I compared to Group II. Additionally, VAS resting scores were also statistically significantly lower in Group I compared to Group III at all time points (p < 0.05). Similarly, the post-hoc test indicated that the VAS coughing scores at the 4^th^ hour (p: 0.023), 12^th^ hour (p: 0.011), and 48^th^ hour (p: 0.019) were statistically significantly lower in Group I compared to Group II. Moreover, VAS coughing scores were statistically significantly lower in Group I compared to Group III at all time points (p < 0.001). Furthermore, there were statistically significant differences in terms of additional analgesic use between the groups (p: 0.001). Additionally, there was a statistically significant difference in terms of morphine consumption via PCA and morphine milligram equivalent consumption between the groups (p < 0.001).

**Conclusions:**

Higher postoperative VAS scores with TPVB applied in obese patients and the consequent increase in additional analgesics and complications require more specific postoperative management in this patient group.

## Background

Thoracic surgery is closely associated with severe pain and if postoperative pain is not controlled, this acute pain may increase the rate of postoperative pulmonary complications (atelectasis, infections), hinder recovery, and even lead to chronic pain [1–4]. Compared to open thoracotomy procedures, video-assisted thoracoscopic surgery (VATS) provides significant advantages such as less postoperative pain, better postoperative pulmonary function, lower mortality and morbidity, and shorter hospital stay [4–7]. Significant pain is a common occurrence, although lower postoperative pain levels are expected with VATS. The intercostal nerve injuries, rib contractions or even fractures, muscle injuries, and pleural lining damage all contribute to pain after thoracoscopic surgery [6, 8]. As a result of all this, it is very important to control the resulting pain. Inadequate acute pain control causes increased postoperative pulmonary complications, chronic pain, and decreased patient satisfaction [6, 9].

Various regional analgesia techniques such as thoracic paravertebral block (TPVB) in combination with standard parenteral/oral drugs have been used for a long time for postoperative analgesia after VATS [6, 10, 11]. TPVB; it also has a lower potential for side effects, besides providing similar analgesia compared to thoracic epidural analgesia (TEA) [6, 10–12]. Also, less side effects are seen because it causes unilateral somatosensory and sympathetic block [12]. Enhanced recovery after surgery (ERAS) and European Society of Regional Anaesthesia (ESRA) also recommend TPVB for acute pain after VATS [5, 13, 14].

Obesity is defined by the World Health Organization as a state of excessive or abnormal fat accumulation that can increase health risks [15]. Obesity and its accompanying comorbidities significantly increase the risk of perioperative surgical complications. Although complications are mostly related to the respiratory system, increased block failures and peripheral nerve injuries are also common [16]. In addition to the difficulty of block applications in obese patients, there is no evidence-based recommendation for the dose of local anesthetic to be used in peripheral nerve block [17, 18]. There is no clear consensus on the relationship between obesity and pain. Some studies suggest that obese patients may experience more pain, while others indicate that they might have a higher pain threshold [19, 20]. As a result, the pain threshold can vary between obese and non-obese individuals, with both increased and decreased pain thresholds observed in obese patients compared to non-obese individuals [21–23].

The hypothesis of this study is that as the body mass index (BMI) increases, the postoperative pain of the patients may increase and the standard analgesia treatment applied may be insufficient. In this study, it was aimed to investigate the effects of BMI on postoperative pain scores in patients who underwent TPVB with 30 ml of 0.25% bupivacaine for postoperative analgesia after VATS.

## Materials and methods

### Study design and patients

The study was conducted in two centers (Ankara Bilkent City Hospital and Ankara Atatürk Sanatorium Training and Research Hospital) as a prospective interventional study design after obtaining approval from Ankara Bilkent City Hospital Institutional Review Board (IRB: E.Kurul-E1-22-2596 / 20/04/2022). The study was published at Clinicaltrials.gov (NCT05357976 / 03/05/2022) after ethics committee approval (principal investigator: Nilgün ZENGİN, MD). All attempts and practices were carried out within the framework of ethical rules and in accordance with the Declaration of Helsinki.

The study included the patients who underwent elective VATS, were in the age range of 18 to 80 years, were assigned American Society of Anesthesiologists (ASA) physical status classifications of I–III were with a BMI of 18–40 kg/m^2^. Patients who did not agree to participate in the study, had chronic pain, had a history of chronic opioid use, were operated under emergency conditions, converted from VATS to thoracotomy, had bleeding disorders, had infection at the injection site, or allergy to local anesthetics were excluded from the study.

Patients were informed about the study, and their written consent was obtained. During the preoperative evaluation, the patients were informed about pain assessment and patient-controlled analgesia (PCA).

The patients were divided into 3 groups according to their BMI.

- ***Group I***: Patients with a BMI of 18-24.9 kg/m^2^.
- ***Group II***: Patients with a BMI of 25-29.9 kg/m^2^.
- ***Group III***: Patients with a BMI of 30–40 kg/m^2^.

### Outcomes

In this study, the primary outcome was determined as visual analog scale (VAS) scores at rest and cough. Secondary outcomes were determined as postoperative morphine consumption, additional analgesic requirement, and side effects.

The standard anesthesia and analgesia protocols described in the following paragraphs were applied to all patients.

### Anesthesia and analgesia protocols

As the authors, we apply the following protocols for general anesthesia and multimodal analgesia in VATS applications in our institutions (similar protocols have also been published in our previous studies) [24–27].

### General anesthesia

In the operating room patients were monitored in accordance with the ASA standards. Patients were administered 0.03 mg/kg midazolam for premedication. After the preoxygenation, anesthesia was induced with 2 mg/kg propofol, 1.5 mcg/kg fentanyl, and 0.1 mg/kg vecuronium. Intubation was performed with a left-sided double-lumen endobronchial tube. An arterial blood pressure monitoring was performed. Anesthesia was maintained by administering sevoflurane in oxygen and air mixture and by administering remifentanil infusion at a dose of 0.01–0.20 mcg/kg/min. Biportal VATS was applied to the patients and a single chest tube was inserted.

### Block procedures

Block procedures were performed under general anesthesia. The blocks were performed under ultrasound (US)-guidance when patients were in the lateral decubitus position. In all patients, a linear probe in a sterile cover was placed 2–3 cm laterally to the spinous process of the fifth thoracic (T5) vertebrae. A US-compatible 22-Gauge and 8-mm nerve block needle was used. The needle was advanced via the in-plane technique until reaching the paravertebral space. A volume of 30 ml of 0.25% bupivacaine was injected into the area and pleural depression was observed.

The block applications were applied to all patients by two anesthetists who had experience with obese patients and were certified (in March 2015) and experienced in the use of US in both centers.

### Analgesia protocol

In the perioperative period, multimodal analgesia was achieved by administering 100 mg of tramadol and 50 mg of dexketoprofen intravenously at the end of the surgery. Additionally, 10 mg of metoclopramide was given to prevent nausea and vomiting. During the postoperative period, patients received 50 mg dexketoprofen every 12 h and 1 g paracetamol every 8 h for continued pain management. Intravenous PCA with morphine was also administered for 24 h. The PCA pump was programmed to deliver a bolus dose of 1 mg morphine and a maximum total dose of 12 mg morphine within 4 h, with lockout intervals of 15 min. Pain levels were assessed using a VAS ranging from 0 (no pain) to 10 (unbearable pain). If the VAS at rest (VASr) score was ≥ 4, rescue analgesia was provided by administering 0.5 mg/kg of tramadol intravenously. Patients were closely monitored in the surgical intensive care unit for 24 h and then transferred to the ward. From the second day, the patients received tramadol 50 mg capsules every 8 h, paracetamol 500 mg tablets, and dexketoprofen 25 mg tablets every 12 h. VAS scores at rest and while coughing were recorded in the postoperative 1^st^, 2^nd^, 4^th^, 12^th^, 24^th^, and 48^th^ hours. Additionally, the need for additional analgesics and side effects (such as; allergic reactions, respiratory depression, urinary retention, nausea-vomiting, and itching) were documented. All patients’ BMI, age, gender, diagnosis, type of surgery, hemodynamic data, intraoperative and postoperative complications, postoperative VAS scores, and postoperative additional analgesic use were recorded. VAS follow-ups were conducted by pain management nurses who were not part of the study.

### Statistical analysis and sample size

Data analyses were conducted using SPSS for Windows, version 22.0 (SPSS Inc., Chicago, IL, United States). The normality of continuous variables was assessed using the Kolmogorov-Smirnov test. The Levene test was employed to evaluate the homogeneity of variances. Continuous data were presented as mean ± standard deviation (SD) for normally distributed variables and as median (Q1: first quartile – Q3: third quartile) for skewed distributions. Categorical data were reported as the number of cases and percentages (%). To compare differences in normally distributed variables among three independent groups, the One-Way ANOVA test was utilized. The Kruskal-Wallis test was applied for comparisons involving non-normally distributed data. The Conover-Iman test was used for binary comparisons among the groups. For categorical variables, Pearson’s chi-square test or Fisher’s exact test was employed as appropriate. A p-value of < 0.05 was considered statistically significant for all analyses.

The sample size for the present study was determined using G*Power© software version 3.1.9.2, developed by the Institute of Experimental Psychology, Heinrich Heine University in Dusseldorf, Germany. The statistical test chosen for the main hypothesis (VAS scores while resting in the first postoperative hour) was the Kruskal-Wallis test. Based on pilot study results and considering a two-tailed (two-sided) type I error rate of 0.05 and a power of 95% (1-β = 0.95), the effect size (d) factor was estimated to be 0.52. With these parameters, the calculated sample size for the study was determined to be ≥ 63 subjects.

## Results

**Fig. 1:**
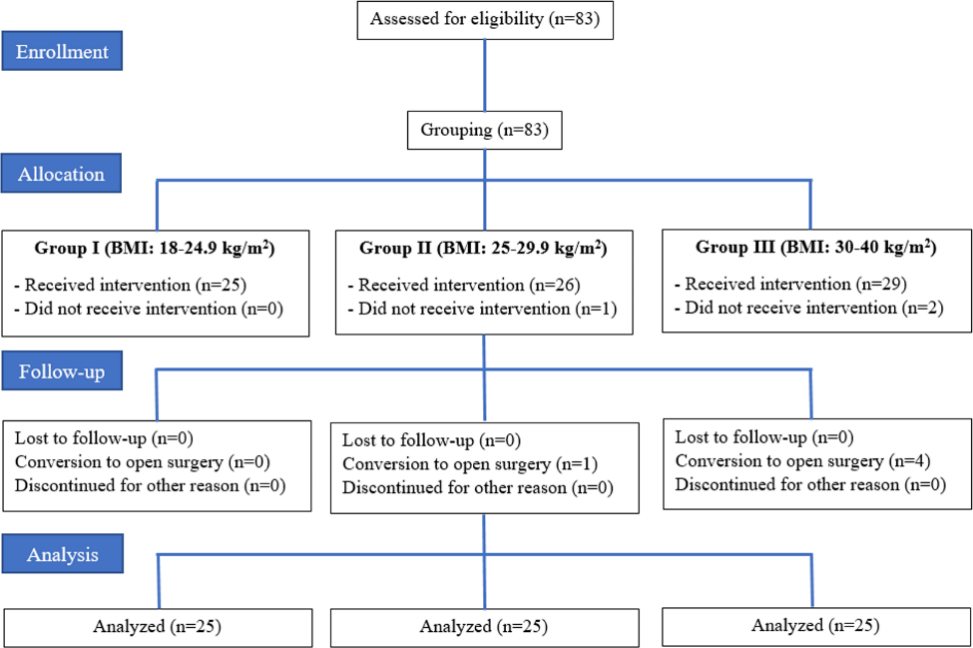
Flowcharts of the patients

The study was conducted between April 2022 and May 2023. 83 patients were included in the study. The patients were divided into 3 groups according to their BMI. TPVB could not be performed on 1 patient in Group II and 2 patients in Group III. In addition, conversion from VATS to thoracotomy occurred in 1 patient in Group II and 4 patients in Group III. Totally, 8 patients were excluded from the study and 75 patients completed the study (Fig. 1).

Demographic and surgical characteristics of the patients are shown in Table 1.

**Table 1:** Demographic and surgical characteristics of the patients

		Group I (n:25)	Group II (n:25)	Group III (n:25)	*p*
**Age (years)** *		51.84 ± 18.39	54.4 ± 12.06	57.08 ± 12.41	0.447
**Gender** ^*#*^	Female	7 (28.0%)	9 (36.0%)	12 (48.0%)	0.380
	Male	18 (72.0%)	16 (64.0%)	13 (52.0%)	
**BMI, kg/m** ^**2***§*^		21.2 (19.8–23.8)	26.2 (25.5–28.6)	31.7 (30.9–34.3)	**< 0.001** ^**a,b,c**^
**Diagnosis** ^*#*^	Pulmonary Nodule	20 (80.0%)	25 (100.0%)	24 (96.0%)	**0.042** ^**a,b**^
	Pneumothorax	5 (20.0%)	-	1 (4.0%)	
**Surgery** ^*#*^	Wedge Resection	16 (64.0%)	19 (76.0%)	15 (60.0%)	0.646
	Segmenthectomy	2 (8.0%)	-	2 (8.0%)	
	Lobectomy	7 (28.0%)	6 (24.0%)	8 (32.0%)	
**ASA** ^*#*^	I	1 (4.0%)	2 (8.0%)	-	0.574
	II	9 (36.0%)	10 (40.0%)	13 (52.0%)	
	III	15 (60.0%)	13 (52.0%)	12 (48.0%)	
**Duration (min)** ^*§*^		150 (120–210)	150 (120–200)	180 (150–240)	0.626

*Continuous variables are expressed as either the mean ± standard deviation (SD) or median (Q1-Q3). Categorical variables are expressed as numbers and frequency (percentage). p < 0.05 statistically significant. The Conover-Iman Test was performed for binary comparisons among the groups*

*One-Way ANOVA test; §The Kruskal-Wallis Test; #χ2 test

*Significant differences were found between;****a***: *I vs. II;****b***: *I vs. III;****c***: *II vs. III.*

*BMI: Body Mass Index; ASA: American Society of Anesthesiologists*

The groups were statistically similar in terms of MAP, HR, and SpO_2_ during the postoperative 24 h.

**Fig. 2:**
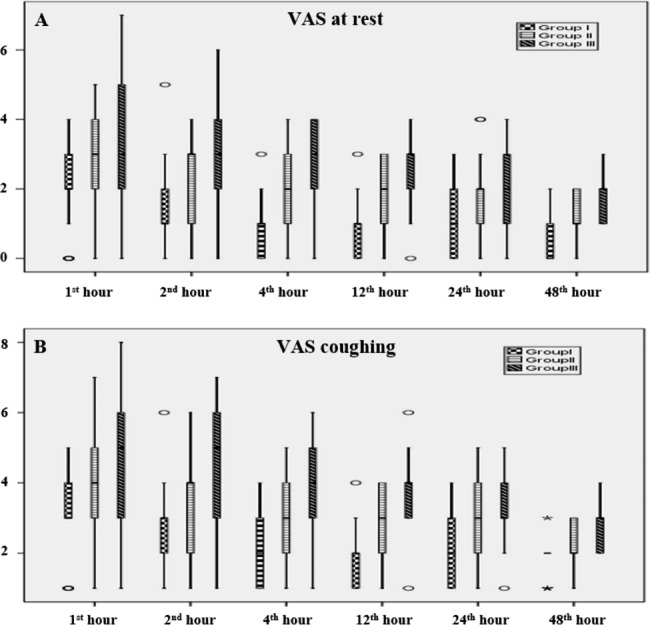
**A**: VAS scores at rest. **B**: VAS scores while coughing. Data are expressed as median (horizontal bar), interquartile range (box), and maximum and minimum values (whiskers) for the VAS scores in the 1st, 2nd, 4th, 12th, 24th, and 48th hours. VAS: Visual analog scale

The groups were compared in terms of VAS resting scores at the 1^st^, 2^nd^, 4^th^, 12^th^, 24^th^, and 48^th^ hours. A statistically significant difference was observed at all time points. Subsequently, a post-hoc test was conducted to determine the source of this difference. VAS resting scores at the 4^th^ hour (p: 0.007), 12^th^ hour (p: 0.014), and 48^th^ hour (p: 0.002) were all statistically significantly lower in Group I compared to Group II. Additionally, VAS resting scores were statistically significantly lower in Group I compared to Group III at all time points (p < 0.05) (Table 2; Fig. 2).

**Table 2:** VAS scores of patients while at rest and coughing

	Group I (n:25)	Group II (n:25)	Group III (n:25)	*p* ^*§*^	Pairwise comparisons (*p*)*				
					a	b		c	
**VAS at rest**									
1^st^ hour	2 (2–3)	3 (2–4)	3 (2–5)	**0.001** ^**b**^	0.066		**0.001**		0.454
2^nd^ hour	1 (1–2)	3 (1–3)	3 (2–4)	**< 0.001** ^**b**^	0.072		**< 0.001**		0.214
4^th^ hour	1 (0–1)	2 (1–3)	3 (2–4)	**< 0.001** ^**a, b**^	**0.007**		**< 0.001**		0.164
12^th^ hour	1 (0–1)	2 (13)	3 (2–3)	**< 0.001** ^**a, b**^	**0.014**		**< 0.001**		0.174
24^th^ hour	1 (0–2)	2 (1–2)	2 (1–3)	**0.003** ^**b**^	0.149		**0.002**		0.479
48^th^ hour	0 (0–1)	1 (1–2)	2 (1–2)	**< 0.001** ^**a, b**^	**0.002**		**< 0.001**		0.396
**VAS coughing**									
1^st^ hour	3 (3–4)	4 (3–5)	5 (3–6)	**< 0.001** ^**b**^	0.066		**< 0.001**		0,353
2^nd^ hour	3 (2–3)	4 (2–4)	5 (3–6)	**< 0.001** ^**b**^	0.091		**< 0.001**		0,173
4^th^ hour	2 (1–3)	3 (2–4)	4 (3–5)	**< 0.001** ^**a, b**^	**0.023**		**< 0.001**		0,088
12^th^ hour	2 (1–2)	3 (2–4)	4 (3–4)	**< 0.001** ^**a, b, c**^	**0.011**		**< 0.001**		**0,035**
24^th^ hour	2 (1–3)	3 (2–4)	3 (3–4)	**0.001** ^**b**^	0.146		**< 0.001**		0,216
48^th^ hour	2 (2–2)	2 (2–3)	3 (2–3)	**< 0.001** ^**a, b**^	**0.019**		**< 0.001**		0,224

Continuous variables are expressed as median (Q1-Q3). p<0.05 statistically significant. The Conover-Iman Test* was performed for binary comparisons among the groups

^*§*^ *The Kruskal-Wallis Test*

*p<0.05 statistically significant*

*Significant differences were found between;****a***: *I vs. II;****b***: *I vs. III;****c***: *II vs. III*

Similarly, the groups were compared in terms of VAS coughing scores at the 1^st^, 2^nd^, 4^th^, 12^th^, 24^th^, and 48^th^ hours. A statistically significant difference was found at all time points. Post-hoc analysis revealed that VAS coughing scores at the 4^th^ hour (p: 0.023), 12^th^ hour (p: 0.011), and 48^th^ hour (p: 0.019) were statistically significantly lower in Group I compared to Group II. Additionally, VAS coughing scores were statistically significantly lower in Group I compared to Group III at all time points (p < 0.05). Notably, VAS coughing scores at the 12^th^ hour were also statistically significantly lower in Group II compared to Group III (Table 2; Fig. 2).

The groups were evaluated in terms of side effects, and only nausea and itching were observed. Nausea was most prevalent in Group III and least in Group I (p: 0.006). Moreover, there was a statistically significant difference between the groups in terms of additional analgesic use (p: 0.001). Group III had the highest use of additional analgesics, while Group I had the lowest. Post-hoc analysis indicated that additional analgesic use was statistically significantly higher in Group III compared to both Group I and Group II (Table 3).

**Table 3:** Morphine consumption via PCA, MME consumption, additional analgesic use, and complication rates

	Group I (n:25)	Group II (n:25)	Group III (n:25)	*p*
**Complication (nausea) n (%)** ^*#*^	-	5 (20.0%)	8 (32.0%)	**0.006** ^**b, c**^
**Complication (ichting) n (%)** ^*#*^	-	1 (4.0%)	3 (12.0%)	0.315
**Additional Analgesic Use n (%)** ^*#*^	1 (4.0%)	8 (32.0%)	13 (52.0%)	**0.001** ^**a,b**^
**Morphine Consumption via PCA (mg)** ^*§*^	7 (5–13)	18 (6–22)	26 (20–32)	**< 0.001** ^**b,c**^
**MME consumption (mg)** ^*§*^	7 (5–13)	18 (12–22)	32 (21–45)	**< 0.001** ^**a,b,c**^

*Continuous variables are expressed as median (Q1-Q3). Categorical variables are expressed as numbers and frequency (percentage). p<0.05 statistically significant. The Conover-Iman Test was performed for binary comparisons among the groups. The P value was set at 0.05*

^*§*^ *The Kruskal-Wallis Test;* ^*#*^ *χ2 test*

*Significant differences were found between;****a***: *I vs. II;****b***: *I vs. III;****c***: *II vs. III.*

*MME*: *Morphine milligram equivalent; PCA: Patient-controlled analgesia*

Furthermore, a statistically significant difference was observed when comparing the groups in terms of morphine consumption via PCA (p < 0.001). Post-hoc analysis indicated that patients in Group III had significantly higher morphine consumption compared to both Group I and Group II (Table 3).

Finally, when the groups were compared in terms of morphine milligram equivalent (MME) consumption, there was a statistically significant difference between the groups (p < 0.001). Post-hoc analysis indicated that MME consumption was higher in Group III while it was lower in Group I (Table 3).

## Discussion

In this study, in which we evaluated the analgesic effectiveness according to BMI in patients who underwent TPVB for postoperative analgesia due to VATS, the increase in BMI caused both an increase in postoperative VAS values and an increase in the need for additional analgesics. Considering the side effects of opioids, the increase in the consumption of additional analgesics and morphine indirectly increases the side effects that may occur due to opioid consumption.

TPVB, which has been actively used in both thoracotomy and VATS in thoracic surgery for the last three decades, has postoperative analgesic results close to or even similar to TEA [28, 29]. Many studies and even meta-analyses show that TPVB can be used as an acceptable method for the management of analgesia after thoracic surgery [5, 12, 13]. While these studies and meta-analyses focused on technique and postoperative analgesic efficacy in TPVB applications, studies on the correlation between BMI and postoperative analgesic effect are quite limited [17]. In our study, we preferred to use a single injection application instead of catheter applications, since VATS applications are less painful than thoracotomy.

Obesity, which is one of the most important challenging situation of the century, is encountered with increasing frequency and has become a serious problem in relation to many morbidities and mortality [30]. In anesthesia applications, the difficulties that may be experienced in the technique to be applied and the conditions arising from the pathophysiological results of obesity can complicate the management of analgesia in this patient group [16]. Central and peripheral blocks, which are indispensable for multimodal analgesia, are frequently used methods. These blocks are very important in terms of reducing opioid consumption and reducing complications that may develop in obese patients with many problems, including respiratory comorbidities [16].

Studies have indicated that fatty tissue in the epidural, especially in the posterior epidural, is not associated with obesity [31, 32]. However, we could not find any study on whether it is associated with obesity, especially considering that nerve structures in the paravertebral area are in adipose tissue. The paravertebral space is a potential space and the absence of a limiting space such as the epidural space may make us think that obesity-related fat tissue increase is a possible condition in this area [33]. In addition, considering that the nerve roots in this area are surrounded by adipose tissue a possible increase in adipose tissue can both affect the volume of the paravertebral area and limit the spread of local anesthetic to the nerve roots [34]. In our study, high VAS scores and an increased need for additional analgesics in patients with high BMI may explain this situation. Radiological and cadaveric studies can be very helpful in clarifying this issue.

The risk of pain following thoracic surgical procedures is significant [35]. Although studies on the relationship between obesity and pain are controversial [36], some research suggests that the intensity of pain is higher in obese patients [19, 21–23]. One such study conducted by Majchrzak et al. [21] emphasized that obese lung cancer patients undergoing thoracic surgery experience higher pain perception compared to nonobese patients, and the duration of severe pain is also longer in obese individuals. In this study, the increase in pain level as BMI increases in patients receiving TPVB in addition to multimodal analgesia supports the results of these studies.

It is undeniable that perioperative management in obese patients brings with it many difficulties. Comorbidities, and especially musculoskeletal disabilities, may present as many sources of pain that are overlooked due to surgery and patient positioning. In these patients, preoperative predisposition to anxiety [37] and its exacerbation with existing pathology may affect postoperative anxiety-related pain [38, 39]. In addition, thick subcutaneous adipose tissue, difficulties in positioning, wider incisions, longer surgery time, and manipulations to reach the surgical site may increase tissue trauma can cause more intense postoperative pain [40, 41]. It can be assumed that more opioids block the severe pain caused by the above-mentioned reasons in this patient group in the postoperative period and this triggering nausea and vomiting may also be a cause of pain by affecting the suture lines. In this study, especially in patients with a BMI above 30 kg/m^2^, the high VAS levels and the high frequency of side effects can be explained by these conditions.

There are some special considerations about the association of obesity with postoperative pain. One of them is genetic polymorphism [42]. Certain genetic polymorphisms may be associated with both obesity and its effects on the body. It has been reported that the number of µ receptors is higher in obese patients due to genetic polymorphism. This polymorphism has been associated with decreased mechanical pain sensitivity and increased requirements for morphine and fentanyl for pain relief [42, 43]. In addition, Dodet et al. [44] reported that the thresholds for detecting cutaneous electrical sensitivity and the pain caused by this sensitivity were significantly higher in obese patients. This sensory dysfunction does not appear to change with weight loss and does not associated with a number of hormonal and genetic factors. Different results on this subject show that more studies are needed to clarify the effect of genetic factors.

Another important factor is that a more exacerbated pain may occur with the addition of the inflammatory response that will occur with surgical trauma to the pro-inflammatory process that is already present in obese patients. It is known that mediators such as interleukin-6, tumor necrosis factor α, and C-reactive protein, which are inflammatory markers, are high in obese patients [45, 46]. The increased amount of adipose tissue in a person can lead to an increased inflammatory response with chemical mediators involved in inflammation, such as prostaglandins, kinins, and histamine, which then interact with the nervous system to produce a sensation of pain, leading to a hyperalgesic process [46]. The addition of different inflammatory mediators, which are caused by the effect of surgery, may increase the level of pain. Our study results also support these definitions.

### Limitations

There are some limitations in this study. There are 6 groups in obesity classification. In this study, patients with BMI < 18 kg/m^2^ and BMI > 40 kg/m^2^ were excluded from the study. In addition, patients with a BMI of 30–40 kg/m^2^ were included in a single group. The reason for this is that the first results are desired to be seen more clearly with fewer groups since there are few publications on this subject in the literature. Therefore, the lack of data for this patient’s groups (BMI < 18 kg/m^2^ and BMI > 40 kg/m^2^) left the question of what kind of clinical situation would be in this patient’s groups unanswered. Secondly, US was performed only by an experienced and certified anesthesiologist in both centers. This does not address the question of what difficulties may be encountered in obese patients when generalizing to anesthesiologists with different training standards in terms of US. Thirdly, although patients with chronic pain treatment and ASA IV were excluded from the study, comorbidities of the patients were not recorded. Fourthly, although acceptable multimodal analgesia protocols were used in both groups, the long lockout period in PCA may limit the 24-hour dosage. Therefore, it may be appropriate to make dose limits to be applied in PCA by considering multimodal analgesia and individual variability. Fifthly, we may have missed the findings at other times, as pain follow-ups were performed at certain times in our study. Finally, since the primary aim of the study was pain levels, the duration of the application, the difficulties encountered during the application, and inflammatory cytokines were not studied.

## Conclusions

In conclusion, pain scores and analgesic requirements were higher in obese patients particularly with obesity class I and obesity class II. Further studies on patients with obesity in class III are needed. In addition, it makes us think that pain may not be related only to the method applied in obese patients and that it is a complex process in which many special conditions such as anatomical, genetic and inflammatory factors caused by obesity in the perioperative period are intertwined. Anatomical, cadaver, radiological, and clinical randomized studies on these issues will be useful in clarifying pain management in obese patients.

## Data Availability

The datasets used and/or analysed during the current study available from the corresponding author on reasonable request.

## Abbreviations

ASA: American Society of Anesthesiologists
BMI: Body mass index
ERAS: Enhanced recovery after surgery
ESRA: European Society of Regional Anaesthesia
HR: Hearth rate
MAP: Mean arterial pressure
MME: Morphine milligram equivalent
SpO_2_: Oxygen saturation
PCA: Patient-controlled analgesia
T: Thoracic
TEA: Thoracic epidural analgesia
TPVB: Thoracic paravertebral block
US: Ultrasound
VAS: Visual analog scale
VATS: Video-assissted thoracoscopic surgery
